# Tertiary lymphoid structures predict the prognosis and immunotherapy response of cholangiocarcinoma

**DOI:** 10.3389/fimmu.2023.1166497

**Published:** 2023-05-10

**Authors:** Taiyu Shang, Tianyi Jiang, Tao Lu, Hui Wang, Xiaowen Cui, Yufei Pan, Mengyou Xu, Mengmiao Pei, Zhiwen Ding, Xiaofan Feng, Yunkai Lin, Xin Li, Yexiong Tan, Feiling Feng, Hui Dong, Hongyang Wang, Liwei Dong

**Affiliations:** ^1^ National Center for Liver Cancer, Naval Medical University, Shanghai, China; ^2^ School of Life Sciences, Institute of Metabolism and Integrative Biology, Fudan University, Shanghai, China; ^3^ Department of Pathology, Eastern Hepatobiliary Surgery Hospital, Naval Medical University, Shanghai, China; ^4^ Department of Hepatobiliary Diseases, Eastern Hepatobiliary Surgery Hospital, Naval Medical University, Shanghai, China; ^5^ Department of Hepatic Surgery, Eastern Hepatobiliary Surgery Hospital, Naval Medical University, Shanghai, China; ^6^ International Cooperation Laboratory on Signal Transduction, Eastern Hepatobiliary Surgery Hospital, Shanghai, China

**Keywords:** cholangiocarcinoma, tertiary lymphoid structures, tumor microenvironment, immune checkpoint inhibitors, prognosis

## Abstract

**Introduction:**

Cholangiocarcinoma (CCA) is a malignant tumor of the biliary epithelium with a poor prognosis. The lack of biomarkers to predict therapeutic response and prognosis is one of the major challenges for CCA treatment. Tertiary lymphoid structures (TLS) provide a local and pivotal microenvironment for tumor immune responses. The prognostic value and clinical relevance of TLS in CCA remain unclear. We aimed to explore the characteristics and clinical significance of TLS in CCA.

**Methods:**

We investigated the prognostic value and clinical relevance of TLS in CCA using a surgery cohort containing 471 CCA patients (cohort 1) and an immunotherapy cohort containing 100 CCA patients (cohort 2). Hematoxylin and eosin (H&E) and immunohistochemical (IHC) staining were used to evaluate the maturity of TLS. Multiplex IHC (mIHC) was employed to characterize the composition of TLS.

**Results:**

Different maturity of TLS were observed in CCA tissue sections. Strong staining of the four-gene signature including PAX5, TCL1A, TNFRSF13C, and CD79A were found in TLS regions. A high density of intra-tumoral TLS (T-score high) were significantly correlated with longer overall survival (OS) both in CCA cohort 1 (p = 0.002) and cohort 2 (p = 0.01), whereas a high density of peri-tumoral TLS (P-score high) were associated with shorter OS in these two cohorts (p = 0.003 and p = 0.03, respectively).

**Conclusion:**

The established four-gene signature efficiently identified the TLS in CCA tissues. The abundance and spatial distribution of TLS were significantly correlated with the prognosis and immune checkpoint inhibitors (ICIs) immunotherapy response of CCA patients. The presence of intra-tumoral TLS are positive prognostic factors for CCA, which provide a theoretical basis for the future diagnosis and treatment of CCA.

## Introduction

Cholangiocarcinoma (CCA) is an epithelial cell malignancy arising from varying locations within the biliary tree, with rising mortality worldwide over the past few decades (1, 2). It can be classified as intrahepatic, perihilar and distal carcinomas in terms of the anatomical location of the tumor in the bile duct tree. Despite the tremendously high postoperative recurrence rate of CCA, surgery remains the prior treatment for patients diagnosed at an early stage. Unfortunately, most CCA patients are afflicted with advanced-stage diseases at initial diagnosis, and neither radiotherapy nor chemotherapy regimens (Gemcitabine and Cisplatin) can significantly improve survival (3). Currently, several studies have attempted to identify the molecular subtypes of CCA and have revealed the critical role of the tumor immune microenvironment (TME) in CCA progression (4–6). Therefore, the direct characterization of TME may contribute to developing novel and effective personalized therapeutic approaches.

A typical histopathological feature of CCA is the presence of abundant stroma, which surrounds and infiltrates tumor structures containing lymphatics, fibrogenic cells, and several immune cells (7). The crosstalk between tumor cells and cells populating the TME contributes to the progression and metastases of CCA. Immunotherapy using antibodies to target immune checkpoints (immune checkpoint inhibitors, ICIs), including the PD-1/PD-L1 and CTLA-4/CD80 pathways, has shown promising anti-cancer effects in a variety of cancers (8–10). Tertiary lymphoid structures (TLS) have been recognized as ectopic aggregated lymphocytes which develop in inflammatory tissues or tumors. TLS is composed of a B-cell zone containing germinal centers and a surrounding T-cell zone comprising several types of T cells, dendritic cells, and high endothelial venules (HEVs) (11, 12). Generally, TLS represents a state of local immune infiltration in the TME since it provides a privileged site for lymphocyte differentiation and antigen presentation, thus providing a crucial environment for both humoral and cellular immune responses against cancer. By modulating immune trafficking and immune response, TLS participates in regulating immune microenvironment and has been associated with better prognosis and elevated immunotherapeutic response in several tumors, such as melanoma, breast cancer, and lung cancer (13–15). However, few studies have demonstrated the prognostic value and immunotherapy relevance of TLS in CCA.

In this study, we aimed to explore the characteristics and clinical significance of TLS in CCA. We investigated and classified TLS in two CCA cohorts, cohort 1 contained 471 cases who received surgery and were treated with standard chemotherapy after postoperative progress, and cohort 2 contained 100 cases who received first-line chemotherapy combined with immune checkpoint inhibitors (ICIs) to prevent postoperative recurrence. Our findings reveal the opposite roles of intra-tumoral and peri-tumoral TLS in predicting the prognosis of CCA and establish new biomarkers for TLS identification.

## Materials and methods

### CCA surgery cohort

The CCA surgery cohort was composed of 471 patients with histologically verified CCA who were surgically resected from 2012 to 2017 at the Eastern Hepatobiliary Surgery Hospital (EHBH), the Naval Medical University, Shanghai, with the approval of the EHBH Research Ethics Committee. All diagnoses were confirmed by pathological analyses. Informed consent was obtained from all patients. The inclusion criteria were as follows: 1) no history of other malignant tumors within 5 years before surgery; 2) no other history of anti-tumor therapy before surgery; 3) between 20 and 75 years of age; 4) no history of immunotherapy and chemotherapy; 5) no perioperative death occurred. The exclusion criteria were: 1) incomplete clinicopathological data; 2) incomplete follow-up information. The data cutoff date for the final analysis was May 3, 2020. Patient characteristics, including age, gender, primary tumor size, primary tumor number, and other tumor parameters relevant to the study, are shown in **Supplementary Table S1**.

### CCA immunotherapy cohort

The CCA immunotherapy cohort was composed of 100 patients who received first-line chemotherapy combined with ICIs to prevent postoperative recurrence from 2017 to 2020 at EHBH, with the approval of the EHBH Research Ethics Committee. All of the patients received R1 and R2 resection, none patients received radical resection. All diagnoses were confirmed by pathological analyses. Patients with Karnofsky Performance Scores (KPS) ≥ 70 were enrolled for further treatment. The first-line chemotherapy was a regimen of GC treatment (1000 mg/m2 gemcitabine and 25 mg/m2 cisplatin in a three-weekly cycle with administrations on days one and eight). The PD-1 inhibitor (anti-PD-1, Sintilimab, Daboshu) is a recombinant fully human monoclonal antibody against programmed death receptor 1, which is independently developed by Innovent Biologics (Suzhou) Co. Ltd. Sintilimab was administered by intravenous infusion at a recommended dose of 200 mg once every three weeks for up to 2 years or until disease progression, intolerable toxicity. Clinical information is shown in **Supplementary Table S2**.

### Evaluation of tumor microenvironment composition

Transcriptomic data were downloaded from the Gene Expression Omnibus (GEO) database (accession code: GSE26566) for a total of 70 samples, including 32 CCA tissues, 32 surrounding liver (SL) tissues, and 6 normal intrahepatic bile duct tissues. The raw microarray data were normalized using R-package *limma* (16). The tumor immune microenvironment (TME) of each sample was then estimated using the MCP-counter tool (17), giving abundance scores of eight immune cells (B cells, T cells, CD8^+^ T cells, NK cells, cytotoxic lymphocytes, neutrophils, myeloid dendritic cells, and monocyte lineage) and two stromal populations (fibroblasts and endothelial cells). Cell composition scores are based on the analysis of specific transcriptomic markers that are specifically and stably expressed in unique cell populations. The relevant characteristics of the cell populations estimated by the MCP counter have been reported in a previous study (18). In addition, the expression of TLS-associated chemokines was explored between CCA and surrounding liver and normal intrahepatic samples. These data sets displayed transcriptional estimates at the gene level, as in log_2_ (x+1) transformed RSEM normalized counts. Genes were mapped to human genome coordinates.

### Differential gene expression analysis of TCGA data, genetic mutation analysis

The mRNA-seq and clinical information of the TCGA CHOL cohort were downloaded from the UCSC Xena platform (http://xena.ucsc.edu/). Data used for tumor genetic mutation analysis were downloaded from cholangiocarcinoma (TCGA, PanCancer Atlas) datasets of cBioPortal (http://www.cbioportal.org/datasets). TLS was quantified using H&E images of the TCGA CHOL cohort (https://portal.gdc.cancer.gov/repository). Tissues containing TLS were considered as “TLS-positive” cases, while tissues with no TLS observed were considered as “TLS-negative” cases. Differential gene analysis was performed between TLS-positive and TLS-negative cases by DESeq2 (version 1.34.0).

### Immunohistochemistry

Formalin-fixed paraffin-embedded tissues were sectioned (4 μm) and stained with hematoxylin and eosin (H&E) for histological analysis or used for immunohistochemistry (IHC). For IHC, endogenous peroxidases were inactivated by 3% hydrogen peroxide and nonspecific signals were blocked by 1% BSA. Sections were incubated with the primary antibody: CD20 (1:200, #MA5-13141, Invitrogen), CD3 (1:200, #ab16669, Abcam), MECA-79 (1:100, #sc-19602, Santa Cruz), CD21 (1:100, #sc-13135, Santa Cruz), CD23 (1:100, #MA5-14572, Invitrogen), TCL1A (1:500, #ab108978, Abcam), PAX5 (1:1000, #ab109443, Abcam), TNFRSF13C (1:300, #ab168389, Abcam), CD79A (1:200, #ab79414, Abcam) at 4°C overnight, HRP-conjugated secondary antibody at 37°C for 1 h, and subsequently stained with DAB substrate. Counterstaining was performed with hematoxylin and mounted with a mounting medium.

### Multiplex immunohistochemistry (mIHC) staining

The slides were dewaxed in xylene, rehydrated through a decreasing ethanol series and fixed in NBF (10% neutral buffered formalin) for 10 min. Slides were stained to enable the simultaneous visualization of four markers: anti-CD23, anti-CD20, anti-CD68, and anti-CD56. At the beginning of each staining cycle, the slides were immersed in Tris-EDTA buffer to perform heat-induced antigen retrieval. After blocking proteins for 10 min, these four primary antibodies were sequentially incubated for 60 min at 37°C. Then the incubation of HRP-conjugated secondary antibody and tyramide signal amplification (TSA) with Opal was followed. Microwave treatment was performed at each cycle of staining to remove the Ab TSA complex. Finally, all slides were stained with 4’-6’-diamidino-2-phenylindole (DAPI, SIGMA-ALDRICH) for 8 min and enclosed with a mounting medium. A full slice scan was performed using Scanner (Pannoramic MIDI, 3DHISTECH).

### Characterization and quantification of TLS

The H&E staining sections of each patient were scanned for whole slide images (WSIs), including tissues from both the tumor and adjacent surrounding liver. TLS was blindly quantified by two pathologists without the knowledge of clinicopathological data. The presence and location of TLS were assessed based on morphology in H&E staining sections. To determine the spatial distribution of TLS, each WSI was subdivided into 2 subregions: intra-tumor (T) and peri-tumor (P) regions. Different abundances and heterogeneous arrangements of TLS were observed in different subregions, and TLS were divided into intra-tumoral TLS and peri-tumoral TLS based on their location to tumor invasive margins. The TLS scoring system was performed as described previously (19). 4 categories for characterization of TLS in the T region (T-score): 1) score 0 for no TLS in the T region (TLS negative CCA); 2) score 1 for 1 or 2 TLS within T region; 3) score 2 for at least 3 TLS in the T region but does not meet the criteria of score 3; 4) score 3 for massive TLS distributed throughout the T region which converges with each other. TLS abundance in the P region (P-score) was scored as follows: 1) score 0 for no TLS in the P region; 2) score 1 for TLS distributed in a localized area of the P region (less than 50%); 3) score 2 for TLS distribution in the majority of the P region (more than 50%); 4) score 3 for massive TLS distributed in the P region (encompassing the entire P region). Granulocytes in necrotic areas were excluded in the assessment. In this study, T-score 0-1 cases were considered as the “T-score low” group, while T-score 2-3 cases were considered as the “T-score high” group. Similarly, P-score 0-1 cases were considered as the “P-score low” group, and P-score 2-3 cases were considered as the “P-score high” group.

TLS maturation stages were investigated using IHC staining. Especially, early TLS contained primary clusters of B and T cells without FDC network and germinal centers (GC centers); primary TLS contained FDC network (CD21) without GC centers; Secondary TLS was identified by CD20^+^ B cells and CD23^+^ FDC cells.

### Statistical analysis

Statistical analyses were performed using SPSS version 21 (IBM, Armonk, NY, USA), GraphPad Prism (version 9.3.0), and Rstudio (version 4.1.3). Categorical variables were compared using Chi-square or Fisher’s exact tests. Kaplan-Meier and log-rank survival analyses were used to compare overall survival (OS) and progression-free survival (PFS). All statistical analyses were two-sided and p-value < 0.05 was considered statistically significant.

## Results

### Immune microenvironment composition of CCA

**Figure 1:**
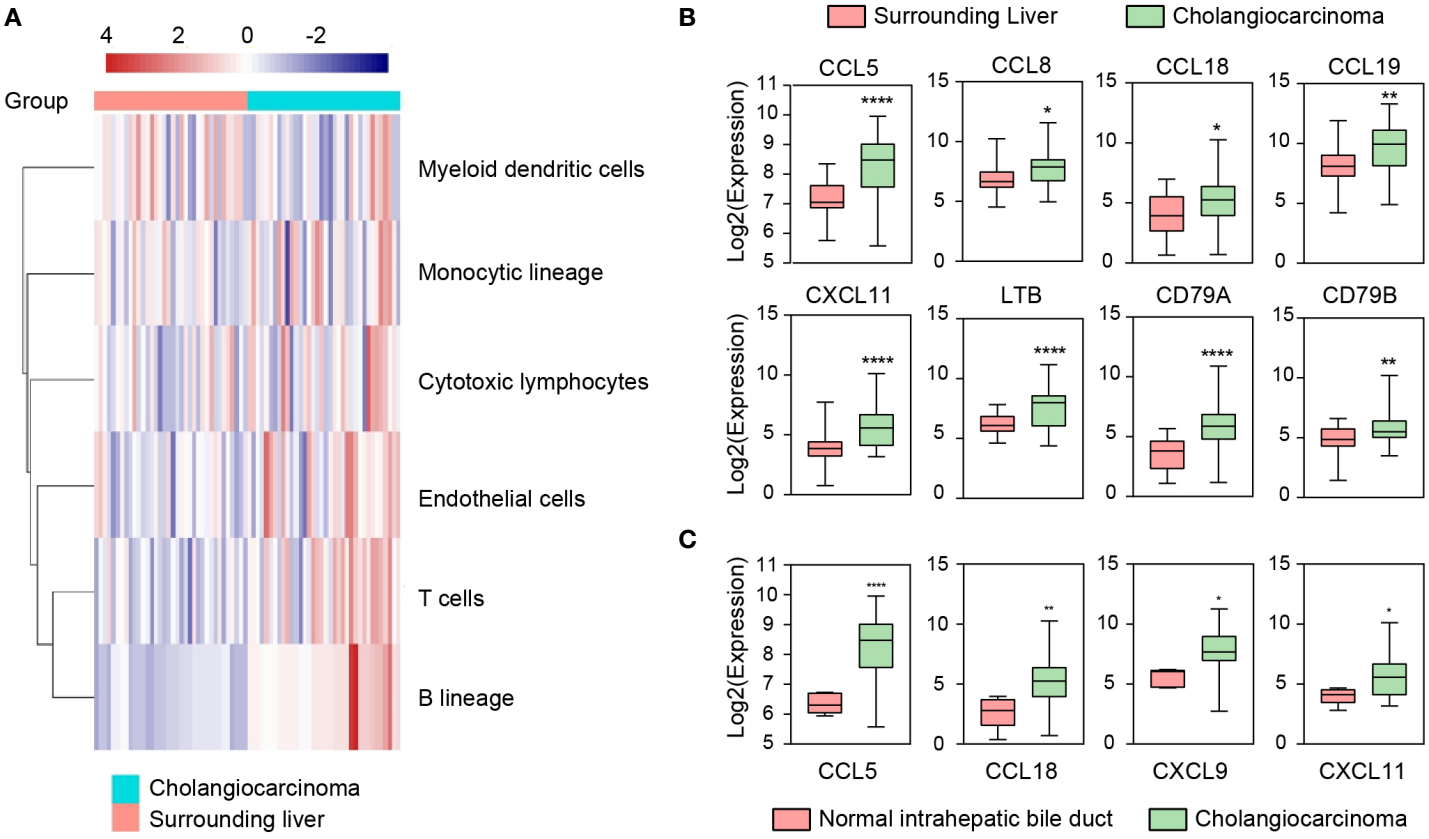
Immune microenvironment (IME) compositions of CCA, surrounding liver, and normal intrahepatic bile duct tissues in GSE26566. **(A)** IME compositions between CCA and surrounding liver tissues detected by MCP-counter. **(B, C)** Expressions of TLS-related factors and chemokines in CCA, surrounding liver, and normal intrahepatic bile duct tissues. Indicators for P values: **** P ≤ 0.0001, ** P ≤ 0.01, * P ≤ 0.05.

We first performed an immune infiltration analysis of 32 CCA and 32 surrounding liver (SL) tissues (GSE26566) with overtly available gene expression profiles by MCP counter. The results showed significant differences in the composition of the immune microenvironment between CCA and SL tissues (**Figure 1A**). Specifically, most samples in the CCA group were enriched with B cells, T cells, monocytic lineage, and cytotoxic lymphocytes, suggesting that TLS may exist. Since multiple chemokines are associated with the presence of TLS, we also investigated the expression of chemokines in the two groups. As expected, chemokines and TLS-related factors including CCL5, CCL8, CCL18, CCL19, CXCL11, LTB, CD79A, and CD79B were remarkably up-regulated in the CCA group compared to the SL group (**Figure 1B**). In addition, higher expression of CCL5, CCL18, CXCL9, and CXCL11 was also detected in CCA tissues compared with the other 6 normal intrahepatic bile duct tissues enrolled in this cohort (**Figure 1C**). In conclusion, these results indicate a high aggregation of tumor-infiltrating immune cells in CCA tissues and also suggest CCA tumor has an immune microenvironment adapted to TLS.

### Different maturity of tertiary lymphoid structures in CCA

**Figure 2:**
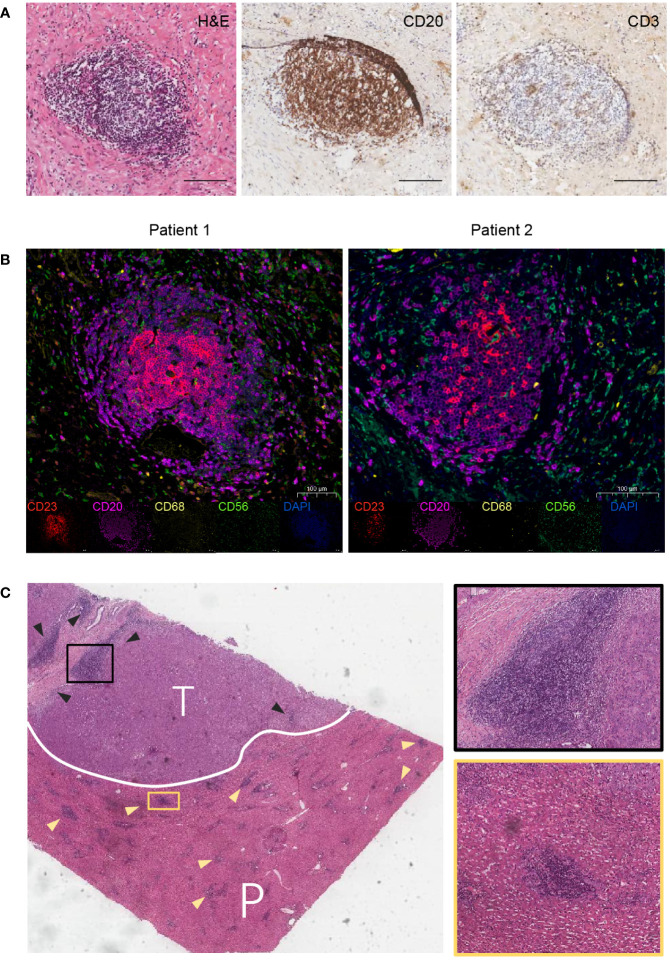
Features of TLS in CCA. **(A)** H&E and IHC staining for a typical TLS in CCA tissue. TLS appeared as clusters of B-cell follicles surrounded by T-cell zones. Scale bars, 100 μm. **(B)** Multiplex immunohistochemistry (mIHC) staining for TLS identification. Markers for follicular dendritic cells (CD23), B cells (CD20), macrophages (CD68), and natural killer cells (CD56) are used. **(C)** H&E staining for intra-tumoral (T) and peri-tumoral (P) regions. Triangles indicate TLS.

Hematoxylin and eosin (H&E) and immunohistochemical (IHC) staining were performed to validate the presence of TLS in CCA tissues. The results showed that TLS consisted of clusters for a CD20^+^ B cell zone and a CD3^+^ T cell zone, with follicular structures formed by B cells as the main TLS component (**Figure 2A**). Then, TLS was identified using multiplex immunohistochemistry staining (mIHC) in tissue sections of CCA patients. The agglomeration of CD23^+^ follicular dendritic cells (FDCs) and CD20^+^ B cells indicated the presence of TLS, along with CD68^+^ macrophages and CD56^+^ natural killer cell (NK cells) sparsely distributed around the aggregates (**Figure 2B**). TLS were found in both intra-tumoral (T) and peri-tumoral (P) tissues in CCA (**Figure 2C**).

**Figure 3:**
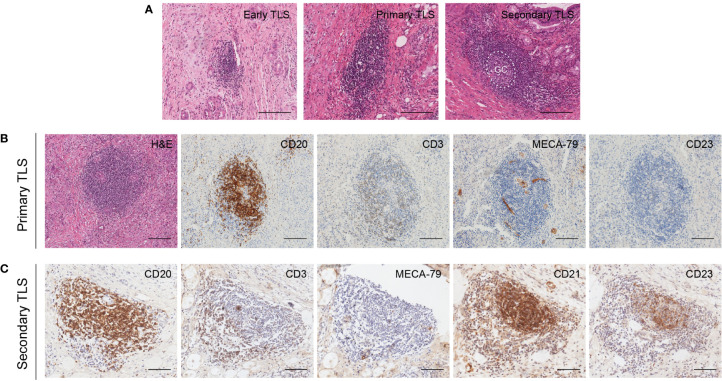
Different maturity and corresponding features of TLS in CCA. **(A)** Different maturity of TLS present in CCA. GC center can be found in the secondary TLS in H&E staining sections. **(B)** Primary TLS. CD20^+^ B cells were diffusely distributed without CD23^+^ follicular structures formation. **(C)** Secondary TLS. CD20^+^ B cells and CD23^+^ FDCs were formed into a follicular structure, with many T cells aggregated around and within the follicles. Scale bars, 200 μm.

TLS has been reported to present in multiple solid tumors and evolves through different stages of maturation (20). Generally, lymphoid aggregates were considered the typical structure of TLS. The presence of multistage maturation of TLS (early TLS, primary TLS, and secondary TLS) could be observed in the H&E-staining sections of CCA (**Figure 3A**). Subsequently, we confirmed the immune cells with cell type-specific surface markers by IHC staining. The results showed that CD20^+^ B cells, CD3^+^ T cells, and MECA-79^+^ HEV were distributed on both primary and secondary TLS in CCA (**Figures 3B, C**). A follicular structure centered on B cells in the network of CD21^+^CD23^+^ FDCs was found in secondary TLS, with multiple T cells clustered around the follicles. In contrast, T cells were dispersed in the TLS without CD23^+^ follicular structures formation, so they were classified as primary TLS.

### Discovery of potential TLS markers for CCA

**Figure 4:**
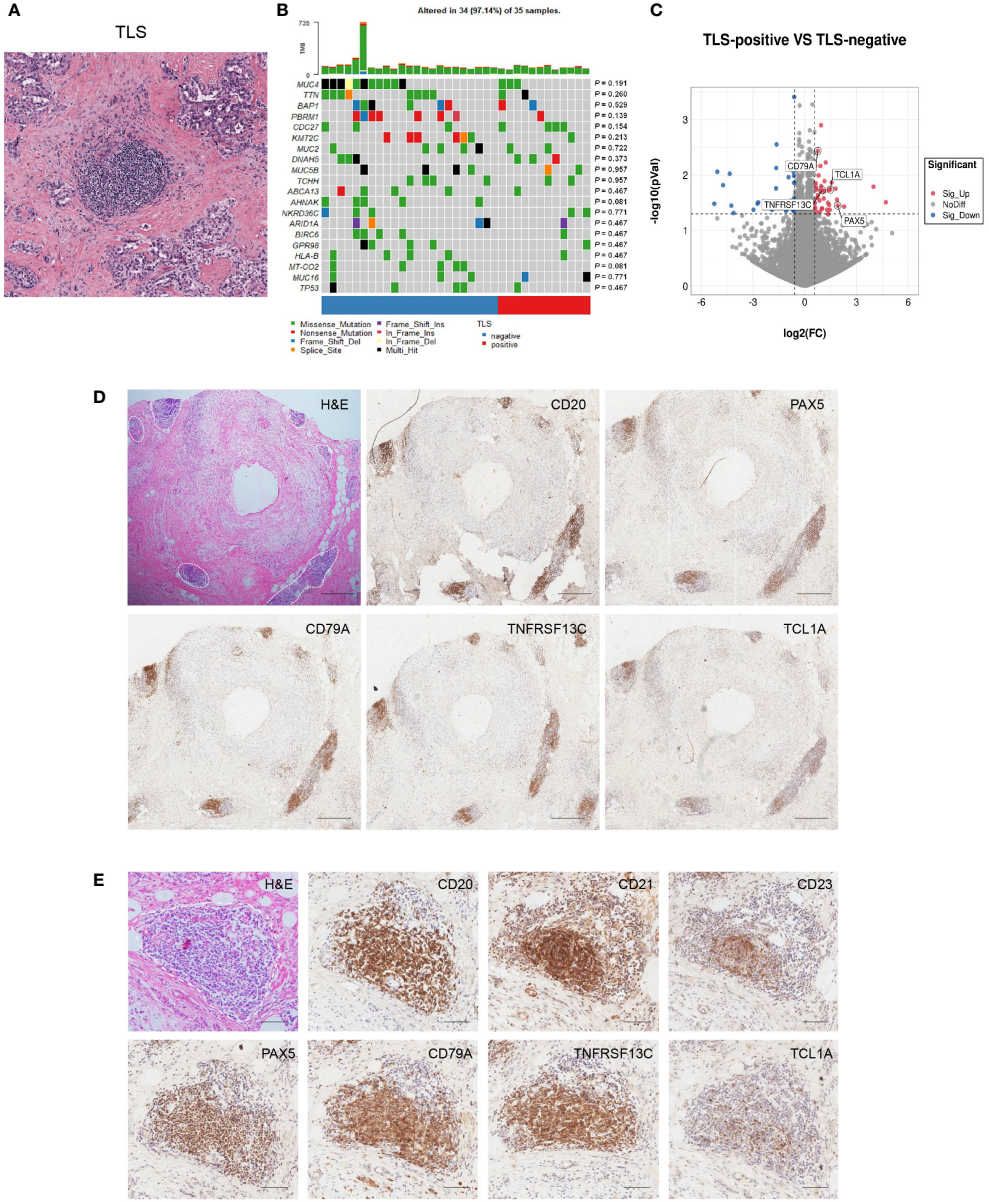
Discovery and validation of four-gene signature for TLS identification. **(A)** Representative H&E image of TLS in CCA. **(B)** Correlation between TLS presence and genetic mutation. The p values were assessed by chi-square tests. **(C)** Differential gene expression of TLS-positive versus TLS-negative CCA cases in the TCGA database. **(D, E)** Representative images of PAX5, TCL1A, TNFRSF13C, and CD79A staining in CCA sections. Scale bars in D, 300 μm. Scale bars in E, 100 μm.

Next, we quantified TLS using H&E-stained tissue sections from the Cancer Genome Atlas (TCGA) cholangiocarcinoma cohort (**Figure 4A**). We divided samples into two groups (TLS-negative group and TLS-positive group) and the relationship between the existence of TLS and genetic mutations was explored. However, no significant difference in genetic mutations was enriched within these two groups harboring distinct TLS status (**Figure 4B**). Consistently, a previous study also showed immune subtypes were irrelevant to genetic mutations (5), suggesting that gene mutation may not be the main factor affecting the presence of TLS in tumors.

To explore the potential markers for TLS, we analyzed the differential gene expression profiles of TLS-positive and TLS-negative CCA cases in the TCGA database. The results showed a higher expression of genes related to B and T cell immunity in the TLS-positive group (**Supplementary Table S3**). The expression of four genes was remarkably elevated in the TLS-positive group: PAX5, TCL1A, TNFRSF13C, and CD79A (**Figure 4C**). We further evaluated the ability of these four molecules to characterize TLS by IHC and found that they all displayed strong staining in the TLS region, indicating a broad application prospect of these four molecules in characterizing TLS (**Figures 4D, E**). These four molecules were also observed in early TLS and primary TLS (**Supplementary Figure 1**).

### TLS in different regions predicts a distinct prognosis of CCA

We next set out to explore the relevance between TLS and clinical features in CCA. A total of 471 CCA patients were enrolled and the clinical characteristics are shown in **Tables 1** and **2**. The median age of the cohort was 56, ranging from 20 to 85 years old, with 160 female patients (34.0%) and 311 male patients (66.0%). The majority of patients had tumors with a diameter of more than 5 cm (303, 64.3%), and a single tumor was observed in most cases (314, 66.7%).

**Table 1:** Correlation analyses between the T-score and the clinicopathological characteristics of 471 CCA patients.

Characteristics		Number n (%)	TLS T-score		χ^2^	P value
			High n (%)	Low n (%)		
Gender	Female	160(34.0)	36(32.7)	124(34.3)	0.099	0.423
	Male	311(66.0)	74(67.3)	237(65.7)		
Age	<65	350(74.3)	86(78.2)	264(73.1)	1.127	0.175
	≥65	121(25.7)	24(21.8)	97(26.9)		
Fatty liver	Negative	411(87.3)	94(85.5)	317(87.8)	0.421	0.308
	Positive	60(12.7)	16(14.5)	44(12.2)		
Tumor size(cm)	<5	168(35.7)	50(45.5)	118(32.7)	5.989	0.010*
	≥5	303(64.3)	60(54.5)	243(67.3)		
Tumor number	Single	314(66.7)	76(69.1)	238(65.9)	0.380	0.310
	Multiple	157(33.3)	34(30.9)	123(34.1)		
Satellite lesions	Absent	311(66.0)	77(70.0)	234(64.8)	1.009	0.187
	Present	160(34.0)	33(30.0)	127(35.2)		
Lymphatic metastasis	Absent	390(82.8)	90(81.8)	300(83.1)	0.098	0.427
	Present	81(17.2)	20(18.2)	61(16.9)		
Distant metastasis	Absent	448(95.1)	104(94.5)	344(95.3)	0.101	0.458
	Present	23(4.9)	6(5.5)	17(4.7)		
Differentiation	Poorly	25(5.3)	7(6.4)	18(5.0)	0.318	0.361
	Well	446(94.7)	103(93.6)	343(95.0)		
Portal vein invasion	Absent	438(93.0)	102(92.7)	336(93.1)	0.016	0.522
	Present	33(7.0)	8(7.3)	25(6.9)		

*P < 0.05

**Table 2:** Correlation analyses between the P-score and the clinicopathological characteristics of 471 CCA patients.

Characteristics		Number n (%)	TLS P-score		χ^2^	P value	
			High n (%)	Low n (%)			
Gender	Female	160(34.0)	63(34.4)	97(33.7)	0.028	0.472
	Male	311(66.0)	120(65.6)	191(66.3)		
Age	<65	350(74.3)	140(76.5)	210(72.9)	0.754	0.224
	≥65	121(25.7)	43(23.5)	78(27.1)		
Fatty liver	Negative	411(87.3)	152(83.1)	259(89.9)	4.752	0.022*
	Positive	60(12.7)	31(16.9)	29(10.1)		
Tumor size(cm)	<5	168(35.7)	68(37.2)	100(34.7)	0.289	0.330
	≥5	303(64.3)	115(62.8)	188(65.3)		
Tumor number	Single	314(66.7)	114(62.3)	200(69.4)	2.574	0.067
	Multiple	157(33.3)	69(37.7)	88(30.6)		
Satellite lesions	Absent	311(66.0)	112(61.2)	199(69.1)	3.110	0.048*
	Present	160(34.0)	71(38.8)	89(30.9)		
Lymphatic metastasis	Absent	390(82.8)	144(78.7)	246(85.4)	3.557	0.040*
	Present	81(17.2)	39(21.3)	42(14.6)		
Distant metastasis	Absent	448(95.1)	172(94.0)	276(95.8)	0.819	0.244
	Present	23(4.9)	11(6.0)	12(4.2)		
Differentiation	Poorly	25(5.3)	8(4.4)	17(5.9)	0.522	0.309
	Well	446(94.7)	175(95.6)	271(94.1)		
Portal vein invasion	Absent	438(93.0)	170(92.9)	268(93.1)	0.004	0.543
	Present	33(7.0)	13(7.1)	20(6.9)		

*P < 0.05

A TLS scoring system was employed to characterize the TLS in this cohort according to the scoring criteria described before. The TLS scores of the intra-tumoral region (T-score) and peri-tumoral region (P-score) were assessed in each case. TLS abundance in intra-tumoral tissues (T-score) was graded into 4 categories, and the TLS in peri-tumoral tissues (P-score) was also scored by another quaternary category. As a result, TLS was found in 438/471 (93.0%) CCA tissues. According to the TLS scoring system, 471 CCA cases could be classified into four groups: T _high_ P _high_ group (Group 1, 31/471, 6.6%), T _high_ P _low_ group (Group 2, 79/471, 16.8%), T _low_ P _high_ group (Group 3, 152/471, 32.3%), and T _low_ P _low_ group (Group 4, 209/471, 44.4%).

The correlations between the TLS score and the clinicopathological characteristics were listed in **Tables 1** and **2**. The results showed that the tumor diameter of the samples with high T-scores was smaller than those with low T-scores (p = 0.010). There were no significant differences in tumor metastasis and differentiated pathology grade between the high T-score and low T-score groups. Patients with high P-score possessed higher incidences of satellite lesions (p = 0.048) and lymphatic metastasis (p = 0.040) than those with low P-score. A higher P-score was also found to be positively associated with fatty liver in patients in this cohort (p = 0.022). Taken together, we discovered that T-score was associated with reduced tumor size, whereas the P-score was associated with a high occurrence of satellite lesions, lymphatic metastasis, and fatty liver (all p < 0.05). These different clinicopathological correlations between T and P scores suggested an essential discrepancy between intra- and peri-tumoral TLS.

**Figure 5:**
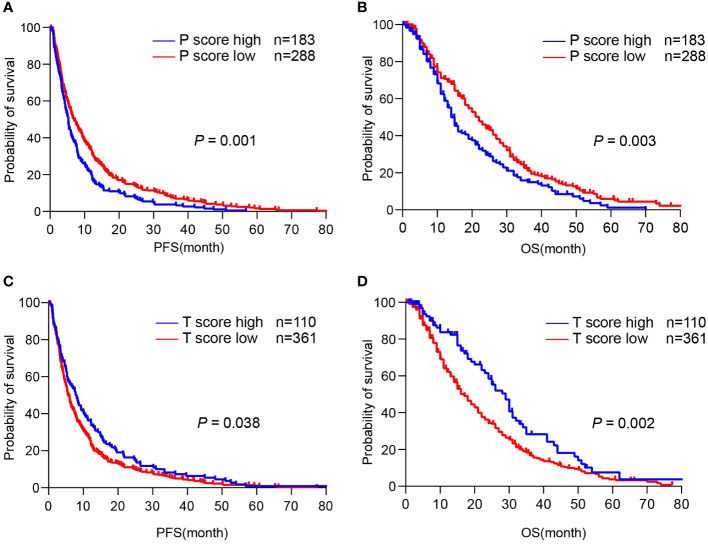
TLS in different regions predicts a distinct prognosis of CCA. **(A, B)** Kaplan-Meier analyses of PFS and OS according to P-score in CCA surgery cohort (n = 471). **(C, D)** Kaplan-Meier analyses of PFS and OS according to T-score in CCA surgery cohort. P values were determined by log-rank tests. OS, overall survival; PFS, progression-free survival.

Next, we asked whether TLS score affected the survival of CCA patients. Since TLS in different locations showed distinct functions, we assessed the predictive effects of the T-score and P-score, respectively. We found that a higher P-score was significantly related to reduced PFS (p = 0.001) and OS (p = 0.003) in CCA patients, as the median PFS was 5 months (95% CI: 4.562-5.438) and the median OS was 14 months (95% CI: 12.103-15.897) in the P-score high group, while the median PFS was 6 months (95% CI: 4.845-7.155) and the median OS was 22 months (95% CI: 19.036-24.964) in the P-score low group (**Figures 5A, B**). In contrast, a higher T-score was significantly related to increased PFS (p = 0.038) and OS (p = 0.002), as the median PFS was 7.567 months (95% CI: 5.454-9.679) and the median OS was 29 months (95% CI: 24.931-33.069) in the T-score high group, whereas the median PFS was 5 months (95% CI: 4.297-5.703) and the median OS was 17 months (95% CI: 14.735-19.265) in the T-score low group (**Figures 5C, D**). These data suggest that a diverse abundance of TLS in different regions predicts a distinct prognosis for CCA patients.

### A high T-score was associated with better prognosis in patients with immune checkpoint inhibitors therapy

**Figure 6:**
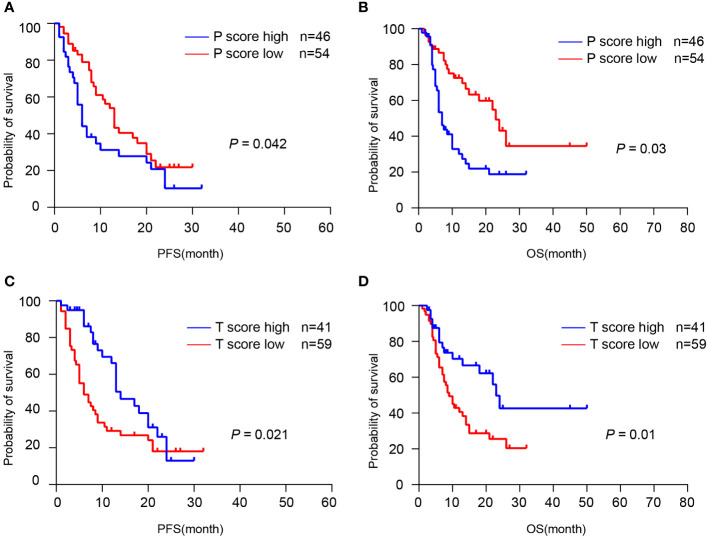
TLS was associated with the response to immunotherapy. **(A, B)** Kaplan-Meier analyses of PFS and OS according to P-score in CCA immunotherapy cohort (n = 100). **(C, D)** Kaplan-Meier analyses of PFS and OS according to T-score in CCA immunotherapy cohort. P values were determined by log-rank tests.

Next, we examined whether TLS can predict the response to ICIs therapy from a cohort of 100 patients who received first-line chemotherapy combined with immune checkpoint inhibitors (ICIs) to prevent postoperative recurrence. H&E staining and IHC staining were performed and the TLS scores were evaluated in the resected tumor sections. Here we discovered that patients in the P-score high group had a relatively shorter PFS (p = 0.042) and OS (p = 0.003) than those in the P-score low group, with a median PFS of 5 months (95% CI: 3.734-6.266) and a median OS of 7 months (95% CI: 5.621-8.379) (**Figures 6A, B**). However, patients in the T-score high group had a significantly longer PFS (p = 0.021) and OS (p = 0.010) compared to those in the T-score low group, with a median PFS of 14 months (95% CI: 10.038-17.962) and a median OS of 23 months (95% CI: 19.917-26.083) (**Figures 6C, D**). The expression of four-gene TLS signature was found not to correlate with patient survival, and there was no significant relevance between tumor cell PDL1 expression and the spatial distribution and abundance of TLS (**Supplementary Figures 2**, **3**). In summary, these data indicate that a high T-score of pre-treatment tumor tissues predicts a better prognosis in patients with immunotherapy, as the presence of intra-tumoral TLS was associated with a prolonged OS and PFS.

## Discussion

CCA are aggressive tumors characterized by highly connective tissue hyperplasia and genetic heterogeneity with a high risk of morbidity and mortality. Available evidence suggests that TME plays a crucial role in CCA progression and metastasis (21, 22). Targeting the components of TME or the crosstalk between CCA cells and TME may create a novel therapeutic approach (23). As an ectopic lymphoid structure, TLS has been detected in multiple tumors and has shown favorable prognostic values (24, 25). Therefore, TLS may be a practicable biomarker to assess the prognosis and immunotherapeutic efficacy of CCA.

By performing the immune infiltration analysis from the GSE26566 database using MCP-counter, a high level of immune cell infiltration was observed in CCA, accompanied by a remarkable up-regulation of chemokines associated with TLS. Then we assessed the features of TLS in CCA sections by H&E and IHC staining. Consistent with previous reports, we found different maturity of TLS presented in CCA such as immature TLS, primary TLS, and secondary TLS. For immature TLS, it is still unknown whether these lymphoid aggregates can participate in further TLS maturation or only remain in an immature state. Fully mature TLS has been reported to contain CD21^+^CD23^+^ FDCs, which distinguishes them from immature or primary TLS (20). Antigen presentation and maturation of B cells may occur in the mature TLS in CCA, leading to the immune response *in situ*. Indeed, the participation of TLS within anti-tumor immune responses has been reported in recent studies (13, 26, 27).

By quantifying TLS using H&E-stained sections from the TCGA cholangiocarcinoma cohort, we found that 28.9% (13/45) cases contained significant clusters of lymphoid aggregates, some of which contained GC centers, which we defined here as “TLS-positive” samples. We further explored the correlation of TLS with high-prevalent genetic mutations, but the results showed no significant relevance, which was consistent with a previous report (5). However, considering the limited number of cases available for analysis, the role of genetic mutations in tumor immunity and TLS maturation requires further investigation. Through profiling the differential gene expression in TLS-negative and -positive groups, we identified four genes, including TNFRSF13C, PAX5, CD79A, and TCL1A, as potential molecules for TLS identification. Among them, TNFRSF13C and PAX5 were associated with B cell lineage commitment (28, 29), and PAX5 also reportedly participated in CCA progression (30). CD79A was suggested to be a marker of activated B cells (31). TCL1A was considered an oncogene, whose up-regulation was observed in B cell malignancies (32). However, the anti-tumor role of TCL1A has also been reported recently (33). In our study, these molecules displayed strong staining in the TLS regions, indicating the feasibility of using them to characterize TLS in CCA. Whether these genes influence the presence and maturation of TLS is currently unknown, but this four-gene signature may contribute to TLS identification by combining with other available markers of TLS.

Studies have shown that TLS is associated with longer overall survival and serves as a prognostic marker in pancreatic cancer and colorectal cancer (34, 35). Our study revealed the prognostic role of TLS based on spatial localization and density in CCA patients. Intra-tumoral TLS is a potent predictor of good prognosis, whereas the presence of peri-tumoral TLS is associated with poor prognosis in CCA. This discrepancy of TLS has been verified in hepatocellular carcinoma and breast cancer (36–38), but the underlying mechanisms remain unclear.

Recent studies have shown that B cells and TLS significantly affected the efficacy of immune checkpoint inhibitors (ICIs). Specifically, the presence of TLS along with B cells in the tissues of non-small cell lung cancer (39), melanoma (13), and urothelial carcinoma (40) increased the efficacy of ICIs. Our result confirmed that intra-tumoral TLS is usually associated with better efficacy of anti-PD-1 treatment. In contrast, a high abundance of peri-tumoral TLS is associated with low immune response and poor prognosis. The different maturation and function of TLS located in different regions in CCA may contribute to this discrepancy. A reliable explanation is that B cells and TLS in the tumor microenvironment were complex, which would exert pro- or anti-tumorigenic functions depending on their maturity to secret antibodies, regulate T cell functions, or present antigens. In immature TLS, B cells secrete molecules that inhibit immune responses and reduce interactions with T cells. In contrast, mature TLS may activate T cells, release antibodies, and further induce antibody-dependent cellular cytotoxicity (ADCC) of tumor cells (20). Actually, intra-tumoral TLS were usually tended to oval-shaped with well-formed maturation, while the peri-tumoral TLS usually displayed as squished, slender, or simply lymphatic aggregates in our H&E staining sections.

In summary, our study established a four-gene TLS signature as practicable biomarker for TLS identification and demonstrated that the spatial distribution and abundance of TLS profoundly affect the prognosis and the immunotherapy response in CCA, providing new perspectives for TLS function and possible clinical intervention.

## Data availability statement

The original contributions presented in the study are included in the article/**Supplementary Material**. Further inquiries can be directed to the corresponding author.

## Ethics statement

Written informed consent was obtained from the individual(s) for the publication of any potentially identifiable images or data included in this article.

## Author contributions

TS, TJ, LD, and HYW designed the study, directed the study, and wrote the manuscript. TS, TJ, TL, and HW performed most of the experiments. YP, MX, MP, ZD, XF, XC, YL, XL, and YT intellectually contributed throughout the project. FF, HD, HYW, and LD guided the project, revised the manuscript, and obtained funding. All authors contributed to the article and approved the submitted version.

## Supplementary material

Supplementary Figure 1 IHC staining of four-gene signature in early TLS and primary TLS. Scale bars, 100 μm.

Supplementary Figure 2 The correlation between patient survival and expression of four-gene signature in the CCA immunotherapy cohort. **(A, B)** Kaplan-Meier analyses of PFS and OS according to PAX5-score in CCA immunotherapy cohort (n = 58). **(C, D)** Kaplan-Meier analyses of PFS and OS according to CD79A-score in CCA immunotherapy cohort (n = 58). **(E, F)** Kaplan-Meier analyses of PFS and OS according to TNFRSF13C-score in CCA immunotherapy cohort (n = 58). **(G, H)** Kaplan-Meier analyses of PFS and OS according to TCL1A-score in CCA immunotherapy cohort (n = 58).

Supplementary Figure 3
**(A)** The correlation between TLS score and expression of four-gene signature in the CCA immunotherapy cohort (n=58). **(B)** The correlation between TLS score and expression of PDL1 in the CCA immunotherapy cohort (n=58).
