# Supplementary figures and images for: Tertiary lymphoid structures predict the prognosis and immunotherapy response of cholangiocarcinoma
Source: Front Immunol. 2023 May 10;14:1166497. doi: 10.3389/fimmu.2023.1166497 (PMC10206168; doi:10.3389/fimmu.2023.1166497)

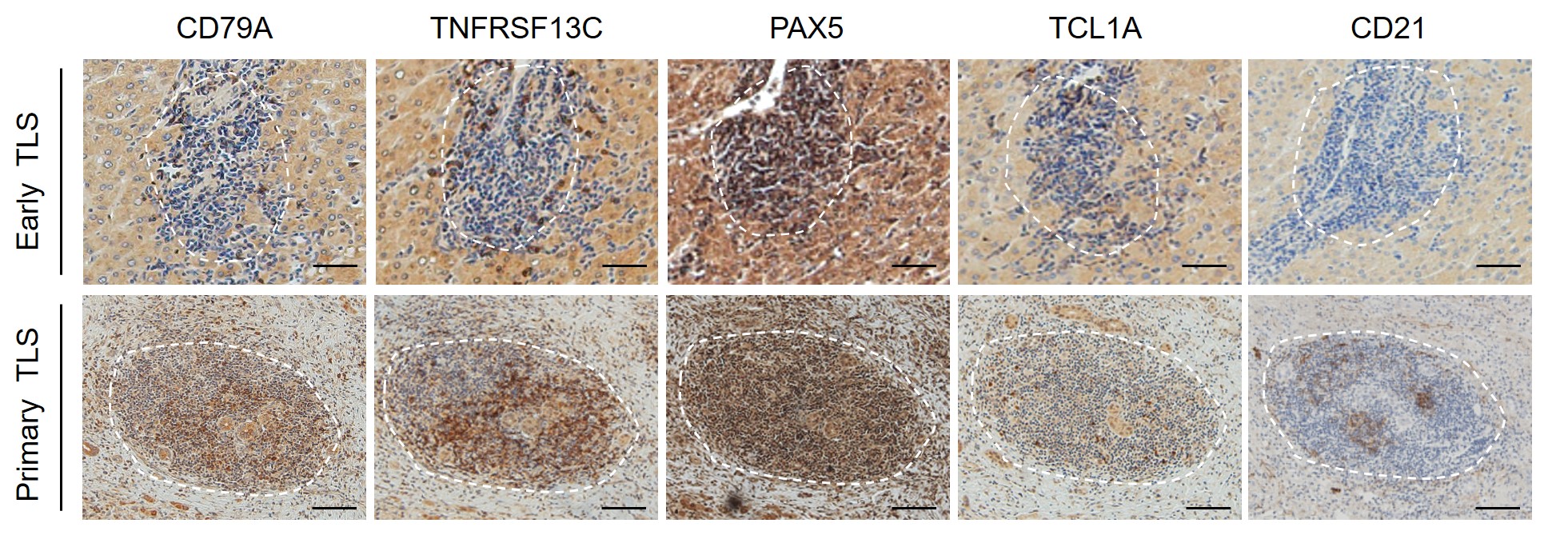

Supplement: Supplementary Figure 1 — IHC staining of four-gene signature in early TLS and primary TLS. Scale bars, 100 μm. [file Image_1.jpeg]

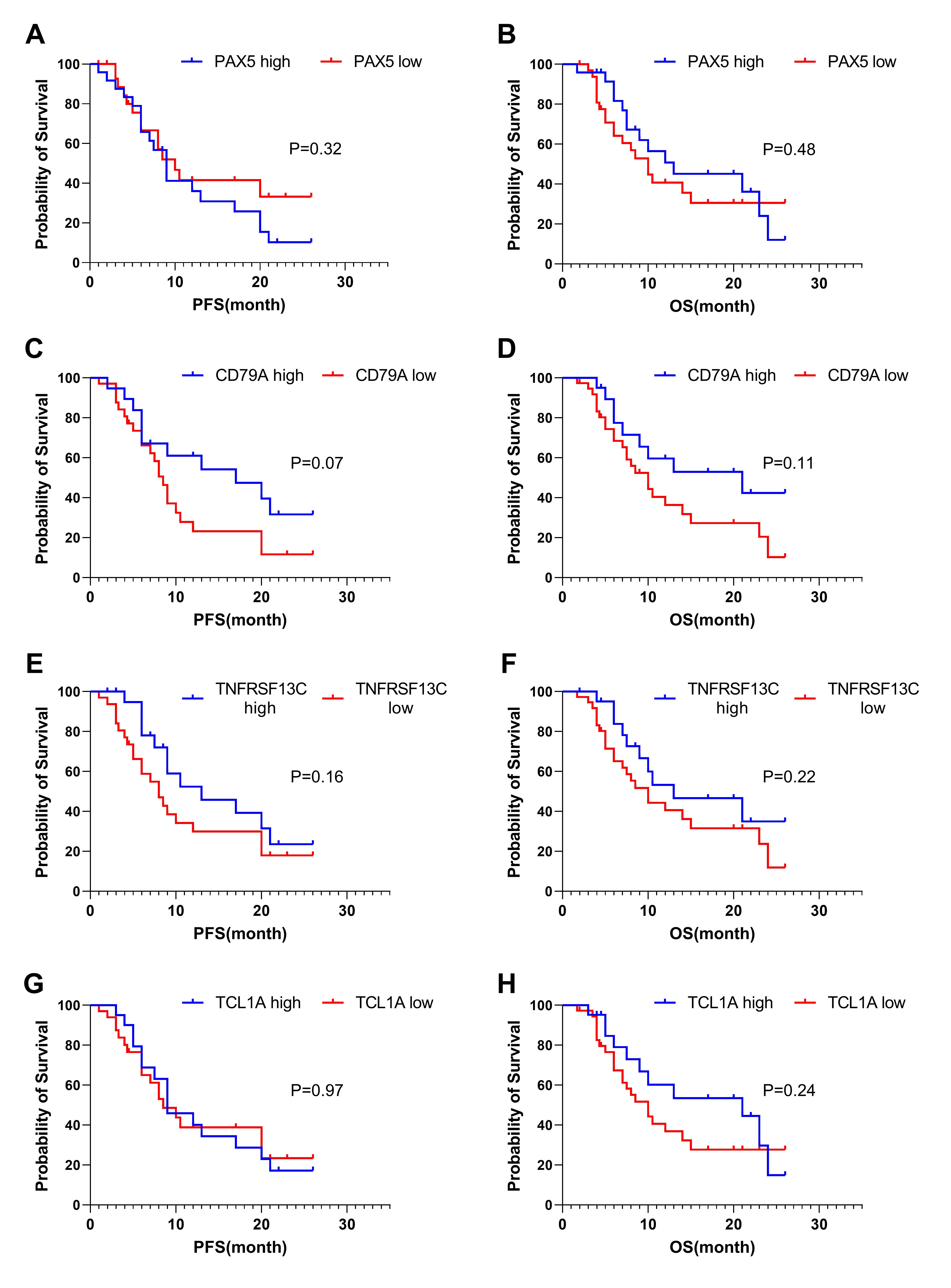

Supplement: Supplementary Figure 2 — The correlation between patient survival and expression of four-gene signature in the CCA immunotherapy cohort. (A, B) Kaplan-Meier analyses of PFS and OS according to PAX5-score in CCA immunotherapy cohort (n = 58). (C, D) Kaplan-Meier analyses of PFS and OS according to CD79A-score in CCA immunotherapy cohort (n = 58). (E, F) Kaplan-Meier analyses of PFS and OS according to TNFRSF13C-score in CCA immunotherapy cohort (n = 58). (G, H) Kaplan-Meier analyses of PFS and OS according to TCL1A-score in CCA immunotherapy cohort (n = 58). [file Image_2.jpeg]

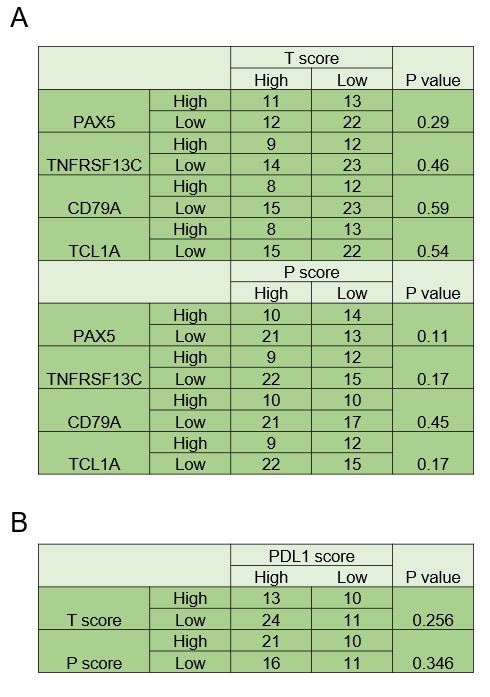

Supplement: Supplementary Figure 3 — (A) The correlation between TLS score and expression of four-gene signature in the CCA immunotherapy cohort (n=58). (B) The correlation between TLS score and expression of PDL1 in the CCA immunotherapy cohort (n=58). [file Image_3.jpeg]
